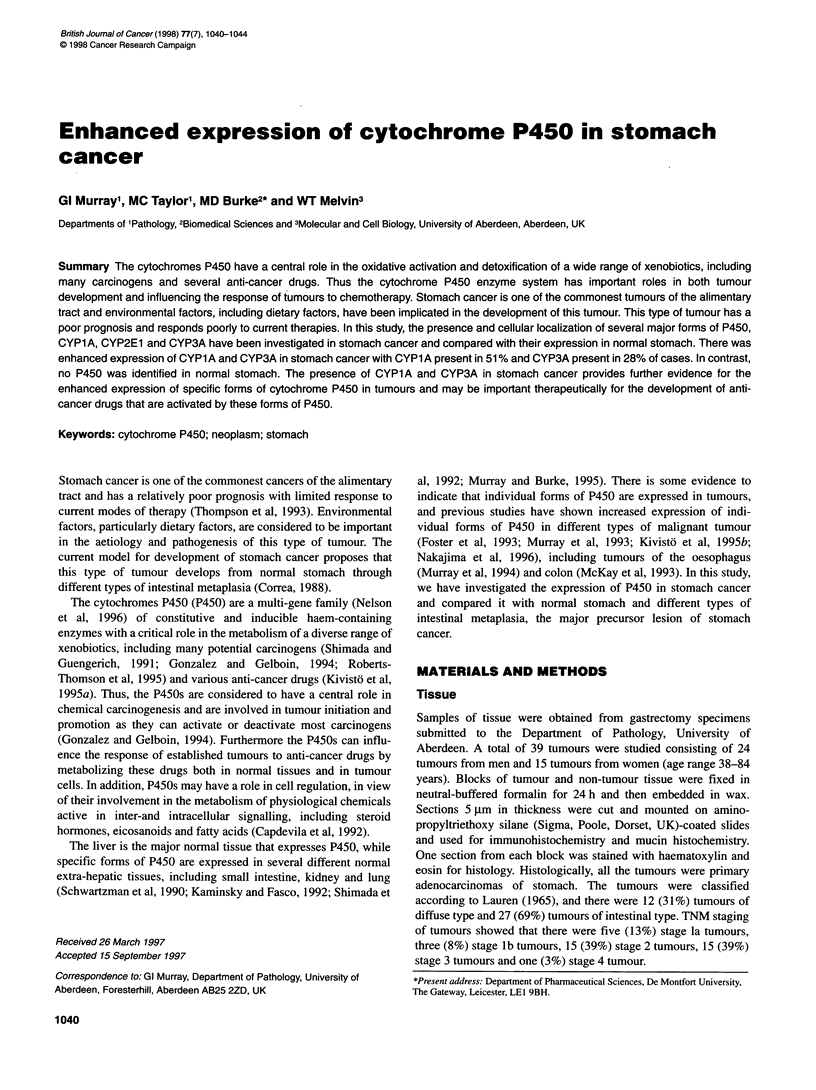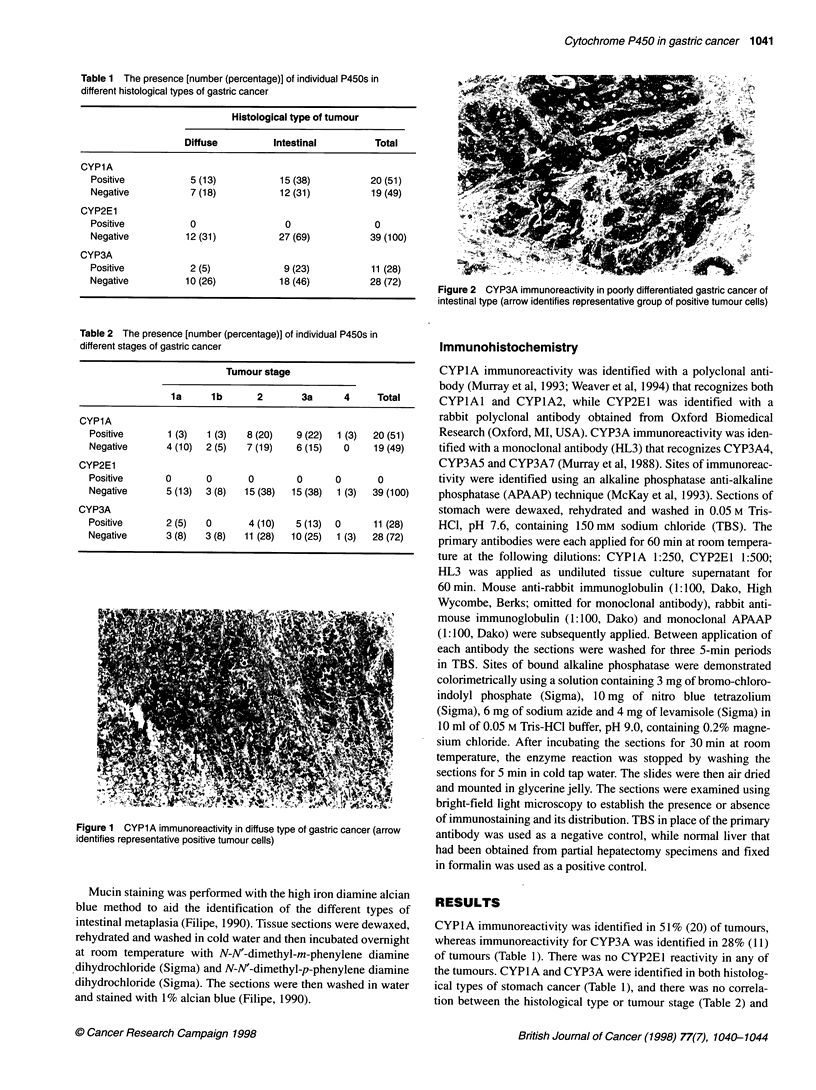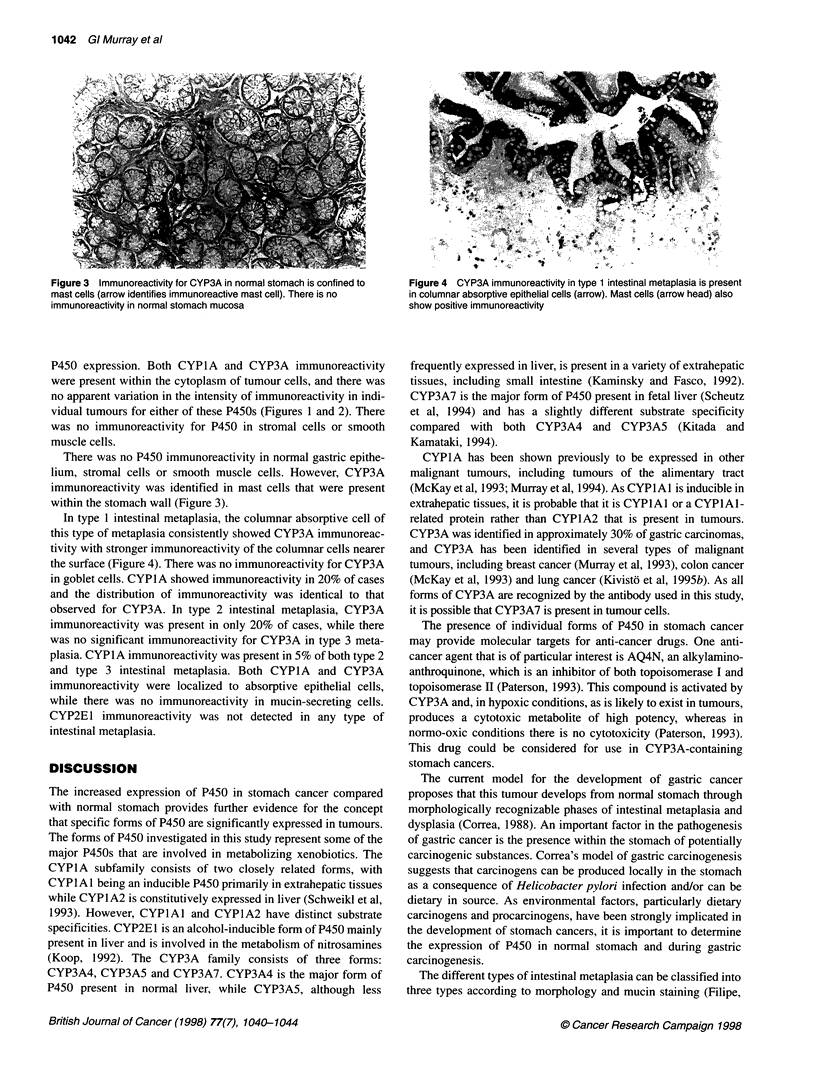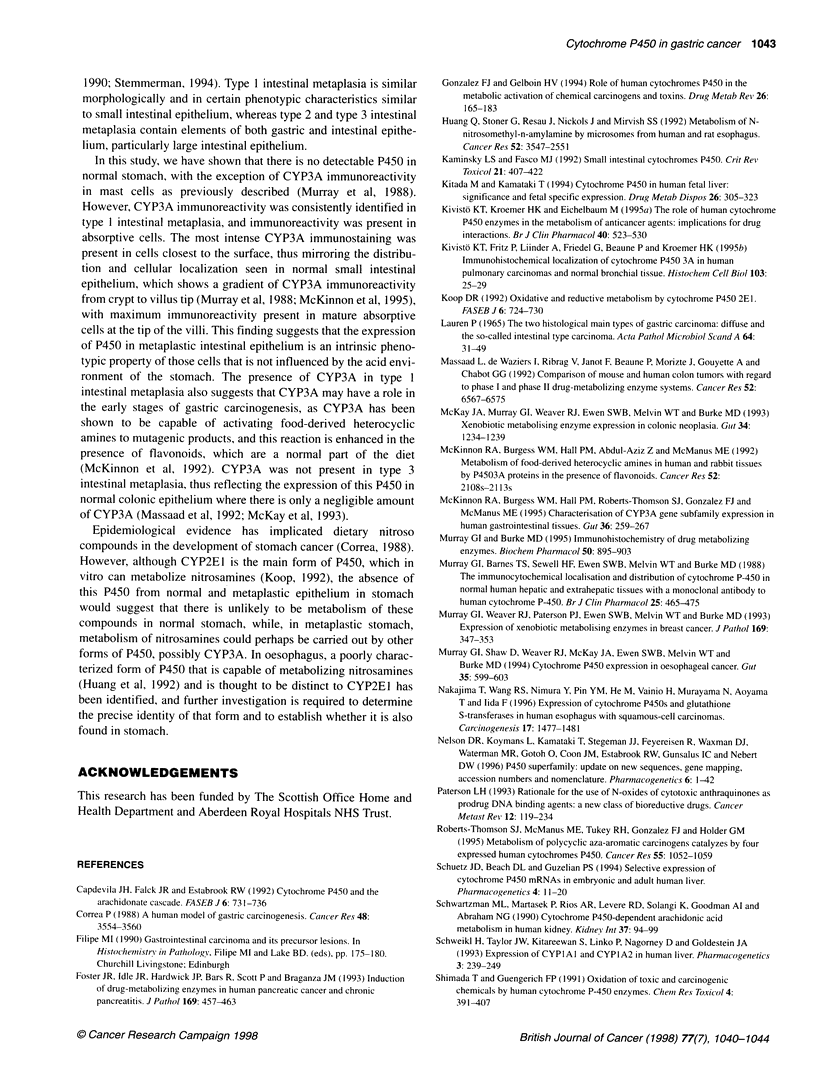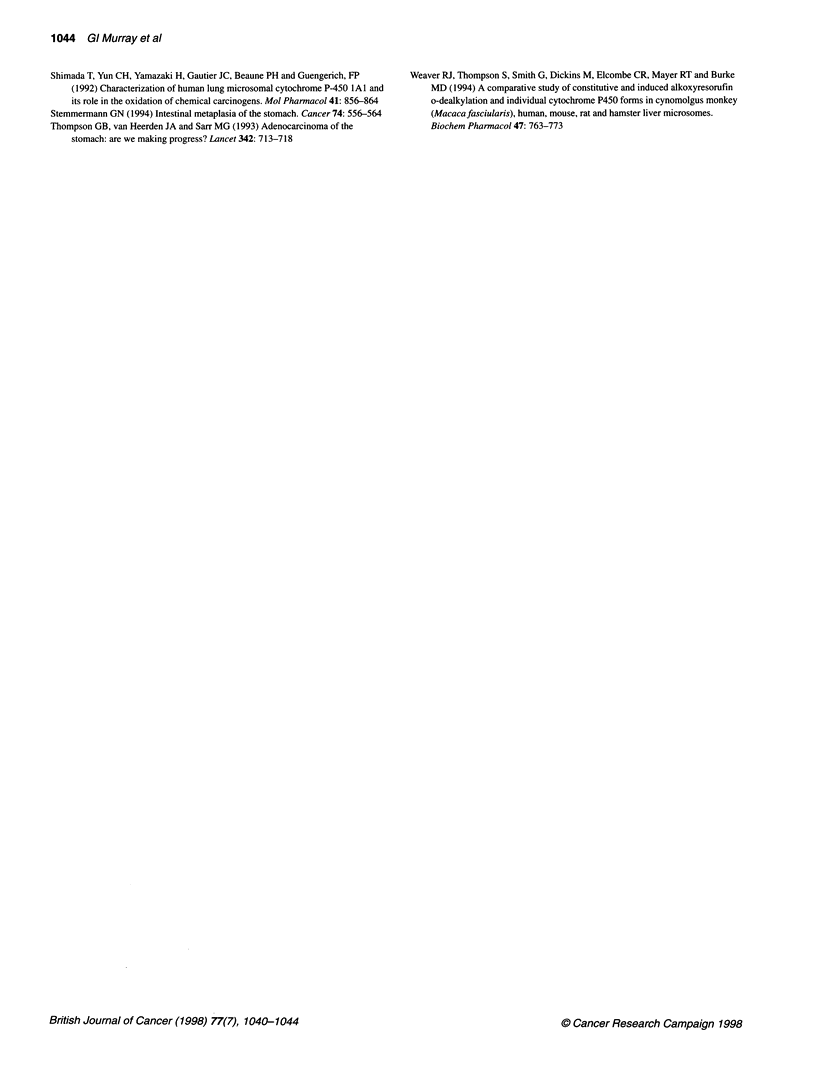# Enhanced expression of cytochrome P450 in stomach cancer.

**DOI:** 10.1038/bjc.1998.173

**Published:** 1998-04

**Authors:** G. I. Murray, M. C. Taylor, M. D. Burke, W. T. Melvin

**Affiliations:** Department of Pathology, University of Aberdeen, Foresterhill, UK.

## Abstract

**Images:**


British Journal of Cancer (1998) 77(7), 1040–1044
© 1998 Cancer Research Campaign

### Enhanced expression of cytochrome P450 in stomach cancer

**GI Murray[1], MC Taylor[1], MD Burke[2]* and WT Melvin[3]**

Departments of [1]Pathology, [2]Biomedical Sciences and [3]Molecular and Cell Biology, University of Aberdeen, Aberdeen, UK

**Summary** The cytochromes P450 have a central role in the oxidative activation and detoxification of a wide range of xenobiotics, including many carcinogens and several anti-cancer drugs. Thus the cytochrome P450 enzyme system has important roles in both tumour development and influencing the response of tumours to chemotherapy. Stomach cancer is one of the commonest tumours of the alimentary tract and environmental factors, including dietary factors, have been implicated in the development of this tumour. This type of tumour has a poor prognosis and responds poorly to current therapies. In this study, the presence and cellular localization of several major forms of P450, CYP1A, CYP2E1 and CYP3A have been investigated in stomach cancer and compared with their expression in normal stomach. There was enhanced expression of CYP1A and CYP3A in stomach cancer with CYP1A present in 51% and CYP3A present in 28% of cases. In contrast, no P450 was identified in normal stomach. The presence of CYP1A and CYP3A in stomach cancer provides further evidence for the enhanced expression of specific forms of cytochrome P450 in tumours and may be important therapeutically for the development of anti-cancer drugs that are activated by these forms of P450.

**Keywords:** cytochrome P450; neoplasm; stomach

Stomach cancer is one of the commonest cancers of the alimentary tract and has a relatively poor prognosis with limited response to current modes of therapy (Thompson et al, 1993). Environmental factors, particularly dietary factors, are considered to be important in the aetiology and pathogenesis of this type of tumour. The current model for development of stomach cancer proposes that this type of tumour develops from normal stomach through different types of intestinal metaplasia (Correa, 1988).

The cytochromes P450 (P450) are a multi-gene family (Nelson et al, 1996) of constitutive and inducible haem-containing enzymes with a critical role in the metabolism of a diverse range of xenobiotics, including many potential carcinogens (Shimada and Guengerich, 1991; Gonzalez and Gelboin, 1994; Roberts-Thomson et al, 1995) and various anti-cancer drugs (Kivistö et al, 1995*a*). Thus, the P450s are considered to have a central role in chemical carcinogenesis and are involved in tumour initiation and promotion as they can activate or deactivate most carcinogens (Gonzalez and Gelboin, 1994). Furthermore the P450s can influence the response of established tumours to anti-cancer drugs by metabolizing these drugs both in normal tissues and in tumour cells. In addition, P450s may have a role in cell regulation, in view of their involvement in the metabolism of physiological chemicals active in inter-and intracellular signalling, including steroid hormones, eicosanoids and fatty acids (Capdevila et al, 1992).

The liver is the major normal tissue that expresses P450, while specific forms of P450 are expressed in several different normal extra-hepatic tissues, including small intestine, kidney and lung (Schwartzman et al, 1990; Kaminsky and Fasco, 1992; Shimada et al, 1992; Murray and Burke, 1995). There is some evidence to indicate that individual forms of P450 are expressed in tumours, and previous studies have shown increased expression of individual forms of P450 in different types of malignant tumour (Foster et al, 1993; Murray et al, 1993; Kivistö et al, 1995*b*; Nakajima et al, 1996), including tumours of the oesophagus (Murray et al, 1994) and colon (McKay et al, 1993). In this study, we have investigated the expression of P450 in stomach cancer and compared it with normal stomach and different types of intestinal metaplasia, the major precursor lesion of stomach cancer.

### MATERIALS AND METHODS

### Tissue

Samples of tissue were obtained from gastrectomy specimens submitted to the Department of Pathology, University of Aberdeen. A total of 39 tumours were studied consisting of 24 tumours from men and 15 tumours from women (age range 38–84 years). Blocks of tumour and non-tumour tissue were fixed in neutral-buffered formalin for 24 h and then embedded in wax. Sections 5 µm in thickness were cut and mounted on amino-propyltriethoxy silane (Sigma, Poole, Dorset, UK)-coated slides and used for immunohistochemistry and mucin histochemistry. One section from each block was stained with haematoxylin and eosin for histology. Histologically, all the tumours were primary adenocarcinomas of stomach. The tumours were classified according to Lauren (1965), and there were 12 (31%) tumours of diffuse type and 27 (69%) tumours of intestinal type. TNM staging of tumours showed that there were five (13%) stage Ia tumours, three (8%) stage Ib tumours, 15 (39%) stage 2 tumours, 15 (39%) stage 3 tumours and one (3%) stage 4 tumour.

Received 26 March 1997
Accepted 15 September 1997

Correspondence to: GI Murray, Department of Pathology, University of Aberdeen, Foresterhill, Aberdeen AB25 2ZD, UK

**Present address:* Department of Pharmaceutical Sciences, De Montfort University, The Gateway, Leicester, LE1 9BH.

**Table 1** The presence [number (percentage)] of individual P450s in different histological types of gastric cancer

| | Histological type of tumour | | |
|---|---|---|---|
| | Diffuse | Intestinal | Total |
| CYP1A | | | |
| Positive | 5 (13) | 15 (38) | 20 (51) |
| Negative | 7 (18) | 12 (31) | 19 (49) |
| CYP2E1 | | | |
| Positive | 0 | 0 | 0 |
| Negative | 12 (31) | 27 (69) | 39 (100) |
| CYP3A | | | |
| Positive | 2 (5) | 9 (23) | 11 (28) |
| Negative | 10 (26) | 18 (46) | 28 (72) |

**Table 2** The presence [number (percentage)] of individual P450s in different stages of gastric cancer

| | Tumour stage | | | | | |
|---|---|---|---|---|---|---|
| | 1a | 1b | 2 | 3a | 4 | Total |
| CYP1A | | | | | | |
| Positive | 1 (3) | 1 (3) | 8 (20) | 9 (22) | 1 (3) | 20 (51) |
| Negative | 4 (10) | 2 (5) | 7 (19) | 6 (15) | 0 | 19 (49) |
| CYP2E1 | | | | | | |
| Positive | 0 | 0 | 0 | 0 | 0 | 0 |
| Negative | 5 (13) | 3 (8) | 15 (38) | 15 (38) | 1 (3) | 39 (100) |
| CYP3A | | | | | | |
| Positive | 2 (5) | 0 | 4 (10) | 5 (13) | 0 | 11 (28) |
| Negative | 3 (8) | 3 (8) | 11 (28) | 10 (25) | 1 (3) | 28 (72) |

**Figure 1** CYP1A immunoreactivity in diffuse type of gastric cancer (arrow identifies representative positive tumour cells)

Mucin staining was performed with the high iron diamine alcian blue method to aid the identification of the different types of intestinal metaplasia (Filipe, 1990). Tissue sections were dewaxed, rehydrated and washed in cold water and then incubated overnight at room temperature with *N-N′*-dimethyl-*m*-phenylene diamine dihydrochloride (Sigma) and *N-N′*-dimethyl-*p*-phenylene diamine dihydrochloride (Sigma). The sections were then washed in water and stained with 1% alcian blue (Filipe, 1990).

**Figure 2** CYP3A immunoreactivity in poorly differentiated gastric cancer of intestinal type (arrow identifies representative group of positive tumour cells)

### Immunohistochemistry

CYP1A immunoreactivity was identified with a polyclonal antibody (Murray et al, 1993; Weaver et al, 1994) that recognizes both CYP1A1 and CYP1A2, while CYP2E1 was identified with a rabbit polyclonal antibody obtained from Oxford Biomedical Research (Oxford, MI, USA). CYP3A immunoreactivity was identified with a monoclonal antibody (HL3) that recognizes CYP3A4, CYP3A5 and CYP3A7 (Murray et al, 1988). Sites of immunoreactivity were identified using an alkaline phosphatase anti-alkaline phosphatase (APAAP) technique (McKay et al, 1993). Sections of stomach were dewaxed, rehydrated and washed in 0.05 M Tris-HCl, pH 7.6, containing 150 mM sodium chloride (TBS). The primary antibodies were each applied for 60 min at room temperature at the following dilutions: CYP1A 1:250, CYP2E1 1:500; HL3 was applied as undiluted tissue culture supernatant for 60 min. Mouse anti-rabbit immunoglobulin (1:100, Dako, High Wycombe, Berks; omitted for monoclonal antibody), rabbit anti-mouse immunoglobulin (1:100, Dako) and monoclonal APAAP (1:100, Dako) were subsequently applied. Between application of each antibody the sections were washed for three 5-min periods in TBS. Sites of bound alkaline phosphatase were demonstrated colorimetrically using a solution containing 3 mg of bromo-chloro-indolyl phosphate (Sigma), 10 mg of nitro blue tetrazolium (Sigma), 6 mg of sodium azide and 4 mg of levamisole (Sigma) in 10 ml of 0.05 M Tris-HCl buffer, pH 9.0, containing 0.2% magnesium chloride. After incubating the sections for 30 min at room temperature, the enzyme reaction was stopped by washing the sections for 5 min in cold tap water. The slides were then air dried and mounted in glycerine jelly. The sections were examined using bright-field light microscopy to establish the presence or absence of immunostaining and its distribution. TBS in place of the primary antibody was used as a negative control, while normal liver that had been obtained from partial hepatectomy specimens and fixed in formalin was used as a positive control.

### RESULTS

CYP1A immunoreactivity was identified in 51% (20) of tumours, whereas immunoreactivity for CYP3A was identified in 28% (11) of tumours (Table 1). There was no CYP2E1 reactivity in any of the tumours. CYP1A and CYP3A were identified in both histological types of stomach cancer (Table 1), and there was no correlation between the histological type or tumour stage (Table 2) and

© Cancer Research Campaign 1998

**Figure 3** Immunoreactivity for CYP3A in normal stomach is confined to mast cells (arrow identifies immunoreactive mast cell). There is no immunoreactivity in normal stomach mucosa

**Figure 4** CYP3A immunoreactivity in type 1 intestinal metaplasia is present in columnar absorptive epithelial cells (arrow). Mast cells (arrow head) also show positive immunoreactivity

P450 expression. Both CYP1A and CYP3A immunoreactivity were present within the cytoplasm of tumour cells, and there was no apparent variation in the intensity of immunoreactivity in individual tumours for either of these P450s (Figures 1 and 2). There was no immunoreactivity for P450 in stromal cells or smooth muscle cells.

There was no P450 immunoreactivity in normal gastric epithelium, stromal cells or smooth muscle cells. However, CYP3A immunoreactivity was identified in mast cells that were present within the stomach wall (Figure 3).

In type 1 intestinal metaplasia, the columnar absorptive cell of this type of metaplasia consistently showed CYP3A immunoreactivity with stronger immunoreactivity of the columnar cells nearer the surface (Figure 4). There was no immunoreactivity for CYP3A in goblet cells. CYP1A showed immunoreactivity in 20% of cases and the distribution of immunoreactivity was identical to that observed for CYP3A. In type 2 intestinal metaplasia, CYP3A immunoreactivity was present in only 20% of cases, while there was no significant immunoreactivity for CYP3A in type 3 metaplasia. CYP1A immunoreactivity was present in 5% of both type 2 and type 3 intestinal metaplasia. Both CYP1A and CYP3A immunoreactivity were localized to absorptive epithelial cells, while there was no immunoreactivity in mucin-secreting cells. CYP2E1 immunoreactivity was not detected in any type of intestinal metaplasia.

### DISCUSSION

The increased expression of P450 in stomach cancer compared with normal stomach provides further evidence for the concept that specific forms of P450 are significantly expressed in tumours. The forms of P450 investigated in this study represent some of the major P450s that are involved in metabolizing xenobiotics. The CYP1A subfamily consists of two closely related forms, with CYP1A1 being an inducible P450 primarily in extrahepatic tissues while CYP1A2 is constitutively expressed in liver (Schweikl et al, 1993). However, CYP1A1 and CYP1A2 have distinct substrate specificities. CYP2E1 is an alcohol-inducible form of P450 mainly present in liver and is involved in the metabolism of nitrosamines (Koop, 1992). The CYP3A family consists of three forms: CYP3A4, CYP3A5 and CYP3A7. CYP3A4 is the major form of P450 present in normal liver, while CYP3A5, although less frequently expressed in liver, is present in a variety of extrahepatic tissues, including small intestine (Kaminsky and Fasco, 1992). CYP3A7 is the major form of P450 present in fetal liver (Scheutz et al, 1994) and has a slightly different substrate specificity compared with both CYP3A4 and CYP3A5 (Kitada and Kamataki, 1994).

CYP1A has been shown previously to be expressed in other malignant tumours, including tumours of the alimentary tract (McKay et al, 1993; Murray et al, 1994). As CYP1A1 is inducible in extrahepatic tissues, it is probable that it is CYP1A1 or a CYP1A1-related protein rather than CYP1A2 that is present in tumours. CYP3A was identified in approximately 30% of gastric carcinomas, and CYP3A has been identified in several types of malignant tumours, including breast cancer (Murray et al, 1993), colon cancer (McKay et al, 1993) and lung cancer (Kivistö et al, 1995*b*). As all forms of CYP3A are recognized by the antibody used in this study, it is possible that CYP3A7 is present in tumour cells.

The presence of individual forms of P450 in stomach cancer may provide molecular targets for anti-cancer drugs. One anticancer agent that is of particular interest is AQ4N, an alkylaminoanthroquinone, which is an inhibitor of both topoisomerase I and topoisomerase II (Paterson, 1993). This compound is activated by CYP3A and, in hypoxic conditions, as is likely to exist in tumours, produces a cytotoxic metabolite of high potency, whereas in normo-oxic conditions there is no cytotoxicity (Paterson, 1993). This drug could be considered for use in CYP3A-containing stomach cancers.

The current model for the development of gastric cancer proposes that this tumour develops from normal stomach through morphologically recognizable phases of intestinal metaplasia and dysplasia (Correa, 1988). An important factor in the pathogenesis of gastric cancer is the presence within the stomach of potentially carcinogenic substances. Correa's model of gastric carcinogenesis suggests that carcinogens can be produced locally in the stomach as a consequence of *Helicobacter pylori* infection and/or can be dietary in source. As environmental factors, particularly dietary carcinogens and procarcinogens, have been strongly implicated in the development of stomach cancers, it is important to determine the expression of P450 in normal stomach and during gastric carcinogenesis.

The different types of intestinal metaplasia can be classified into three types according to morphology and mucin staining (Filipe,

© Cancer Research Campaign 1998

1990; Stemmerman, 1994). Type 1 intestinal metaplasia is similar morphologically and in certain phenotypic characteristics similar to small intestinal epithelium, whereas type 2 and type 3 intestinal metaplasia contain elements of both gastric and intestinal epithelium, particularly large intestinal epithelium.

In this study, we have shown that there is no detectable P450 in normal stomach, with the exception of CYP3A immunoreactivity in mast cells as previously described (Murray et al, 1988). However, CYP3A immunoreactivity was consistently identified in type 1 intestinal metaplasia, and immunoreactivity was present in absorptive cells. The most intense CYP3A immunostaining was present in cells closest to the surface, thus mirroring the distribution and cellular localization seen in normal small intestinal epithelium, which shows a gradient of CYP3A immunoreactivity from crypt to villus tip (Murray et al, 1988; McKinnon et al, 1995), with maximum immunoreactivity present in mature absorptive cells at the tip of the villi. This finding suggests that the expression of P450 in metaplastic intestinal epithelium is an intrinsic phenotypic property of those cells that is not influenced by the acid environment of the stomach. The presence of CYP3A in type 1 intestinal metaplasia also suggests that CYP3A may have a role in the early stages of gastric carcinogenesis, as CYP3A has been shown to be capable of activating food-derived heterocyclic amines to mutagenic products, and this reaction is enhanced in the presence of flavonoids, which are a normal part of the diet (McKinnon et al, 1992). CYP3A was not present in type 3 intestinal metaplasia, thus reflecting the expression of this P450 in normal colonic epithelium where there is only a negligible amount of CYP3A (Massaad et al, 1992; McKay et al, 1993).

Epidemiological evidence has implicated dietary nitroso compounds in the development of stomach cancer (Correa, 1988). However, although CYP2E1 is the main form of P450, which in vitro can metabolize nitrosamines (Koop, 1992), the absence of this P450 from normal and metaplastic epithelium in stomach would suggest that there is unlikely to be metabolism of these compounds in normal stomach, while, in metaplastic stomach, metabolism of nitrosamines could perhaps be carried out by other forms of P450, possibly CYP3A. In oesophagus, a poorly characterized form of P450 that is capable of metabolizing nitrosamines (Huang et al, 1992) and is thought to be distinct to CYP2E1 has been identified, and further investigation is required to determine the precise identity of that form and to establish whether it is also found in stomach.

### ACKNOWLEDGEMENTS

This research has been funded by The Scottish Office Home and Health Department and Aberdeen Royal Hospitals NHS Trust.

### REFERENCES

Capdevila JH, Falck JR and Estabrook RW (1992) Cytochrome P450 and the arachidonate cascade. *FASEB J* **6**: 731–736

Correa P (1988) A human model of gastric carcinogenesis. *Cancer Res* **48**: 3554–3560

Filipe MI (1990) Gastrointestinal carcinoma and its precursor lesions. In *Histochemistry in Pathology*, Filipe MI and Lake BD. (eds), pp. 175–180. Churchill Livingstone; Edinburgh

Foster JR, Idle JR, Hardwick JP, Bars R, Scott P and Braganza JM (1993) Induction of drug-metabolizing enzymes in human pancreatic cancer and chronic pancreatitis. *J Pathol* **169**: 457–463

Gonzalez FJ and Gelboin HV (1994) Role of human cytochromes P450 in the metabolic activation of chemical carcinogens and toxins. *Drug Metab Rev* **26**: 165–183

Huang Q, Stoner G, Resau J, Nickols J and Mirvish SS (1992) Metabolism of N-nitrosomethyl-n-amylamine by microsomes from human and rat esophagus. *Cancer Res* **52**: 3547–2551

Kaminsky LS and Fasco MJ (1992) Small intestinal cytochromes P450. *Crit Rev Toxicol* **21**: 407–422

Kitada M and Kamataki T (1994) Cytochrome P450 in human fetal liver: significance and fetal specific expression. *Drug Metab Dispos* **26**: 305–323

Kivistö KT, Kroemer HK and Eichelbaum M (1995*a*) The role of human cytochrome P450 enzymes in the metabolism of anticancer agents: implications for drug interactions. *Br J Clin Pharmacol* **40**: 523–530

Kivistö KT, Fritz P, Liinder A, Friedel G, Beaune P and Kroemer HK (1995*b*) Immunohistochemical localization of cytochrome P450 3A in human pulmonary carcinomas and normal bronchial tissue. *Histochem Cell Biol* **103**: 25–29

Koop DR (1992) Oxidative and reductive metabolism by cytochrome P450 2E1. *FASEB J* **6**: 724–730

Lauren P (1965) The two histological main types of gastric carcinoma: diffuse and the so-called intestinal type carcinoma. *Acta Pathol Microbiol Scand A* **64**: 31–49

Massaad L, de Waziers I, Ribrag V, Janot F, Beaune P, Morizte J, Gouyette A and Chabot GG (1992) Comparison of mouse and human colon tumors with regard to phase I and phase II drug-metabolizing enzyme systems. *Cancer Res* **52**: 6567–6575

McKay JA, Murray GI, Weaver RJ, Ewen SWB, Melvin WT and Burke MD (1993) Xenobiotic metabolising enzyme expression in colonic neoplasia. *Gut* **34**: 1234–1239

McKinnon RA, Burgess WM, Hall PM, Abdul-Aziz Z and McManus ME (1992) Metabolism of food-derived heterocyclic amines in human and rabbit tissues by P4503A proteins in the presence of flavonoids. *Cancer Res* **52**: 2108s–2113s

McKinnon RA, Burgess WM, Hall PM, Roberts-Thomson SJ, Gonzalez FJ and McManus ME (1995) Characterisation of CYP3A gene subfamily expression in human gastrointestinal tissues. *Gut* **36**: 259–267

Murray GI and Burke MD (1995) Immunohistochemistry of drug metabolizing enzymes. *Biochem Pharmacol* **50**: 895–903

Murray GI, Barnes TS, Sewell HF, Ewen SWB, Melvin WT and Burke MD (1988) The immunocytochemical localisation and distribution of cytochrome P-450 in normal human hepatic and extrahepatic tissues with a monoclonal antibody to human cytochrome P-450. *Br J Clin Pharmacol* **25**: 465–475

Murray GI, Weaver RJ, Paterson PJ, Ewen SWB, Melvin WT and Burke MD (1993) Expression of xenobiotic metabolising enzymes in breast cancer. *J Pathol* **169**: 347–353

Murray GI, Shaw D, Weaver RJ, McKay JA, Ewen SWB, Melvin WT and Burke MD (1994) Cytochrome P450 expression in oesophageal cancer. *Gut* **35**: 599–603

Nakajima T, Wang RS, Nimura Y, Pin YM, He M, Vainio H, Murayama N, Aoyama T and Iida F (1996) Expression of cytochrome P450s and glutathione S-transferases in human esophagus with squamous-cell carcinomas. *Carcinogenesis* **17**: 1477–1481

Nelson DR, Koymans L, Kamataki T, Stegeman JJ, Feyereisen R, Waxman DJ, Waterman MR, Gotoh O, Coon JM, Estabrook RW, Gunsalus IC and Nebert DW (1996) P450 superfamily: update on new sequences, gene mapping, accession numbers and nomenclature. *Pharmacogenetics* **6**: 1–42

Paterson LH (1993) Rationale for the use of N-oxides of cytotoxic anthraquinones as prodrug DNA binding agents: a new class of bioreductive drugs. *Cancer Metast Rev* **12**: 119–234

Roberts-Thomson SJ, McManus ME, Tukey RH, Gonzalez FJ and Holder GM (1995) Metabolism of polycyclic aza-aromatic carcinogens catalyzes by four expressed human cytochromes P450. *Cancer Res* **55**: 1052–1059

Schuetz JD, Beach DL and Guzelian PS (1994) Selective expression of cytochrome P450 mRNAs in embryonic and adult human liver. *Pharmacogenetics* **4**: 11–20

Schwartzman ML, Martasek P, Rios AR, Levere RD, Solangi K, Goodman AI and Abraham NG (1990) Cytochrome P450-dependent arachidonic acid metabolism in human kidney. *Kidney Int* **37**: 94–99

Schweikl H, Taylor JW, Kitareewan S, Linko P, Nagorney D and Goldestein JA (1993) Expression of CYP1A1 and CYP1A2 in human liver. *Pharmacogenetics* **3**: 239–249

Shimada T and Guengerich FP (1991) Oxidation of toxic and carcinogenic chemicals by human cytochrome P-450 enzymes. *Chem Res Toxicol* **4**: 391–407

© Cancer Research Campaign 1998

Shimada T, Yun CH, Yamazaki H, Gautier JC, Beaune PH and Guengerich, FP (1992) Characterization of human lung microsomal cytochrome P-450 1A1 and its role in the oxidation of chemical carcinogens. *Mol Pharmacol* **41**: 856–864
Stemmermann GN (1994) Intestinal metaplasia of the stomach. *Cancer* **74**: 556–564
Thompson GB, van Heerden JA and Sarr MG (1993) Adenocarcinoma of the stomach: are we making progress? *Lancet* **342**: 713–718
Weaver RJ, Thompson S, Smith G, Dickins M, Elcombe CR, Mayer RT and Burke MD (1994) A comparative study of constitutive and induced alkoxyresorufin o-dealkylation and individual cytochrome P450 forms in cynomolgus monkey (*Macaca fasciularis*), human, mouse, rat and hamster liver microsomes. *Biochem Pharmacol* **47**: 763–773

© Cancer Research Campaign 1998